# Novel Human Parechovirus, Sri Lanka

**DOI:** 10.3201/eid1601.091105

**Published:** 2010-01

**Authors:** Ngan Thi Kim Pham, Quang Duy Trinh, Sayaka Takanashi, Chandra Abeysekera, Asiri Abeygunawardene, Hideaki Shimizu, Pattara Khamrin, Shoko Okitsu, Masashi Mizuguchi, Hiroshi Ushijima

**Affiliations:** University of Tokyo, Tokyo, Japan (N.T.K. Pham, Q.D. Trinh, S. Takanashi, M. Mizuguchi); University of Peradeniya, Peradeniya, Sri Lanka (C. Abeysekera, A. Abeygunawardene); Kawasaki City Institute of Health, Kanagawa, Japan (H. Shimizu); Aino Health Science Center and Aino University, Tokyo (P. Khamrin, H. Ushijima); Aino Science Center and Aino College, Tokyo (S. Okitsu).

**Keywords:** Parechovirus, HPeV10, enteric infections, gastroenteritis, viruses, Sri Lanka, dispatch

## Abstract

Of 362 fecal samples collected from children with acute gastroenteritis in Sri Lanka during 2005–2006, 30 (8.3%) were positive for human parechovirus (HPeV) by reverse transcription–PCR. A novel HPeV, designated as HPeV10, was identified in 2 samples by sequence analysis of the viral protein 1 gene of the detected HPeVs.

Parechoviruses are small, nonenveloped, positive-sense, single-stranded RNA viruses belonging to the large family of *Picornaviridae*, a highly diverse family of important pathogens of humans and animals. The genus *Parechovirus* is composed of 2 species: *Ljungan virus*, isolated from bank voles (*1*), and *human parechovirus* (HPeV), a frequent human pathogen. The HPeV genome is ≈7.3 kb long and contains a large open reading frame coding for a single polyprotein. The polyprotein is cleaved posttranslationally into 3 structural proteins (viral protein [VP] 0, VP3, and VP1) and 7 nonstructural proteins (2A–2C and 3A–3D) (*2*,*3*).

Previous findings have shown the genetic variability of HPeVs, and the number of newly identified HPeV genotypes has been on the increase (*4*–*6*). To date there have been 9 published HPeV types assigned as types 1–8 and 14 (www.picornaviridae.com/parechovirus/hpev/hpev.htm). We identified a novel HPeV designated as HPeV10 that was detected in the stool samples of children in Sri Lanka who had acute gastroenteritis.

## The Study

We used reverse transcription–PCR to screen 362 fecal samples collected from child inpatients with acute gastroenteritis at a hospital in Kandy, Sri Lanka, during September 2005 through August 2006 for HPeV. Informed consent was obtained from the mothers of all enrolled patients. The study was approved by the University of Peradeniya’s Committee on Research and Ethical Review. Reverse transcription was performed by using random primer, and PCR was conducted by using primers ev22(+) and ev22(–) to amplify a 270-bp PCR product of the 5′ untranslated region (*7*).

For genotyping, samples positive for HPeV by the screening PCR were subjected to a 2-step PCR to amplify the VP1 sequence. The first PCR was done by using 2 newly developed primers, Cap-parEcho-F (5′-TCHACWTGGATGMGRAARAC-3′) and Cap-parEcho-R (5′-TCYARYTCACAYTCYTCYTC-3′), which were designed outside the VP1 region, whereas the nested PCR was performed by using the inner primer pair, VP1-parEchoF1 and VP1-parEchoR1, described by Benschop et al. (*8*). The PCR amplicons of the VP1 gene were purified and sequenced in both directions by using the BigDye Terminator Cycle Sequencing kit (Perkin Elmer-Applied Biosystems, Inc., Foster City, CA, USA). The inner primers for amplification of VP1 gene were used as sequencing primers. The sequence data were collected by an ABI Prism 310 Genetic Analyzer (Perkin Elmer-Applied Biosystems, Inc.).

Comparison analysis of the VP1 sequence was conducted between the obtained HPeV strains and reference HPeV strains of the 9 defined genotypes (HPeV1-8 and HPeV14) available in the GenBank database. The sequence data and the phylogenesis were analyzed by using BioEdit version 7.0.5 (www.mbio.ncsu.edu/BioEdit/bioedit.html). A parsimony analysis was also conducted by using MEGA version 3.1 to determine the evolutionary relationship among studied sequences (*9*). The method was performed using close-neighbor interchange with a random option and with 500 bootstrap repetitions.

Of the 362 samples tested, 30 were positive for HPeV; detection rate was 8.3%. Of these, 12 isolates were selected for amplification and sequencing of the VP1 gene. Ten of the 12 sequenced strains were of known and well-characterized genotypes (genotype 1, 7 samples; genotype 4, 3 samples). These strains were not further analyzed. Two remaining strains (LK-103 and LK-106, accession nos. GQ402515 and GQ402516, respectively) showed VP1 sequences that clustered together with none of the known 9 HPeV genotypes (HPeV1-8, 14) in the phylogenetic analysis (Figure). Nucleotide and amino acid similarities between these 2 strains were 94.4% and 99.5%, respectively (data not shown).

**Figure:**
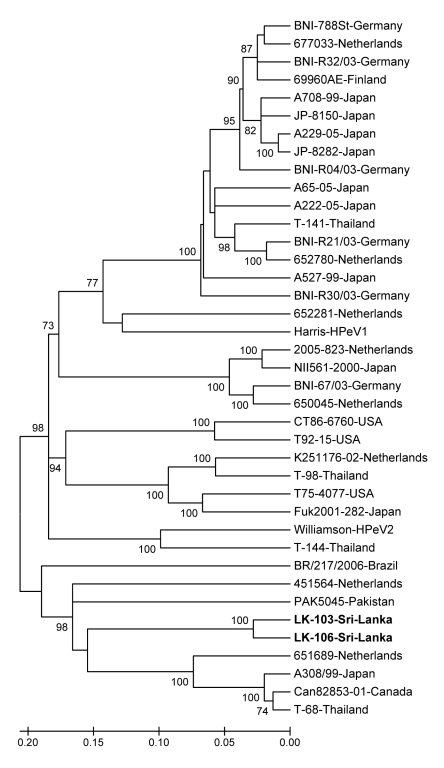
Phylogenetic tree constructed from nucleotide sequences of the structural viral protein 1 gene of the strains studied and reference human parechovirus (HPeV) strains with 500 bootstrap repetitions. Percentage bootstrap values >70% are shown at the branch nodes. The studied HPeV strains are in **boldface**; their nucleotide sequences have been deposited in GenBank under accession nos. GQ402515 and GQ402516. Scale bar indicates nucleotide substitutions per site.

Identical matrix analysis of VP1 nucleotide sequences of the 2 strains from Sri Lanka and global reference strains of the 9 known genotypes available in GenBank databases was then performed. The results showed that the 2 studied strains had highest mean nucleotide and amino acid similarities with HPeV3, 69.1% and 82.8%, respectively, and that the lowest mean nucleotide and amino acid similarities were found between the 2 studied strains and HPeV5, of 54.5% and 60.9%, respectively (Table). Therefore, these strains were expected to be classified into a new or previously unpublished HPeV (HPeV 9-13) genotype according to proposed criteria for assigning HPeV genotypes (*10*).

**Table:** Mean percentage nucleotide similarities between HPeV genotypes*

HPeV genotype	1	2	3	4	5	6	7	8	10	14
1										
2	64.1									
3	58.0	59.0								
4	63. 9	63.2	59.5							
5	61.6	60.7	53.5	65.8						
6	64.7	60.1	61.0	61.8	60.8					
7	57.3	59.9	63.8	58.7	56.0	54.1				
8	61.6	61.7	61.2	62.3	57.7	59.1	62.1			
10	60.5	56.8	69.1	60.9	54.5	55.9	66.0	63.6		
14	59.8	58.4	65.9	58.4	56.2	56.6	66.2	62.5	68.8	

*HPeV, human parechovirus.

The VP1 sequences of the strains studied were submitted to the International Committee on Taxonomy of Viruses Picornavirus Study Group (www.picornastudygroup.com/types/index.html) to identify their genotype. These 2 strains were designated HPeV10 with their nucleotide and amino acid identities of 88.0% and 98.6% (strain LK-106) and 87.7% and 97.7% (strain LK-103) to the prototype BAN2004-10903 (M.S. Oberste et al., unpub. data).

The alignment of deduced amino acid sequences of the strains studied and global HPeV reference strains of HPeVs genotypes 1–8 and 14 showed that the arginine-glycine-aspartic acid (RGD) motif, which is considered to be critical for HPeV1 entry (*11*), was neither present in the strains studied nor among reference strains of HPeV3, HPeV7, HPeV8, and HPeV14 (*4*–*6**,**12*). Therefore, like HPeV3, HPeV7, HPeV8, and HPeV14, the lack of RGD motif in HPeV10 may imply that HPeV10 has an RGD-independent entry pathway.

## Conclusions

We found HPeV in stool samples collected from hospitalized children in Sri Lanka who had acute gastroenteritis. The identified HPeV10 in this study was more closely genetically related to HPeV3 than to the remaining published HPeVs. Together with the unpublished findings of Oberste et al., this study provides basic data for future research into HPeV10. In addition, when taken together with other previous findings, our findings suggest that HPeV should be included in the spectrum of viruses for which routine screening is conducted among children with acute gastroenteritis.
